# Supplementary material for: Using redescription mining to relate clinical and biological characteristics of cognitively impaired and Alzheimer’s disease patients
Source: PLoS One. 2017 Oct 31;12(10):e0187364. doi: 10.1371/journal.pone.0187364 (PMC5663625; doi:10.1371/journal.pone.0187364)
Supplement: S17 File — (PDF) [file pone.0187364.s017.pdf]

Choice of statistical tests

Matej Mihelčić, Tomislav Šmuc
Ruđer Bošković Institute, Zagreb, Croatia
{matej.mihelcic, tomislav.smuc}@irb.hr

Goran Šimić, Mirjana Babić Leko
Department for Neuroscience, Croatian Institute for Brain Research,
University of Zagreb Medical School, Zagreb, Croatia
{gsimic@hiim.hr,mbabic@hiim.hr}

Sašo Džeroski, Nada Lavrač
Jožef Stefan Institute, Ljubljana, Slovenia
{saso.dzeroski, nada.lavrac}@ijs.sl

October 14, 2017

In this document, we motivate our choice of statistical tests used in the manuscript *Using Redescription Mining to Relate Clinical and Biological Characteristics of Cognitively Impaired and Alzheimer's Disease Patients* and shortly describe their assumptions and limitations.

In the manuscript, we perform two different types of statistical tests: a) measuring the difference in distribution between two different subsets of entities and b) measuring the level and statistical significance of correlations between different pairs of attributes.

a) From the tests measuring the difference in distribution between two subsets of entities, we use Mann-Whitney U test [1], Kolmogorov-Smirnov test [2, 3] and Anderson-Darling test [4].

   Mann-Whitney U test is a non-parametric test that can be used without any assumptions on the underlying data distribution to assess if two samples have been obtained from a common distribution. The

one-directional test can be used to determine the shift in distribution. That is, if the null hypothesis of equal distribution is discarded, the alternative hypothesis provides information whether there is higher probability to obtain larger or smaller values in one sample compared to the other. The main assumption of this test is that all observations are independent from each other. The test has been originally designed to work with continuous data (that is no ties should be present), however it has been extended since with the corrections to enable using it on data containing ties.

We use this test in our work to test the difference in distribution of various biological indicators (containing numerical values) between patients with late mild cognitive impairment, Alzheimer's disease and those classified as cognitively normal. The attributes are numerical, however they contain ties due to rounding in measurements and data processing. Since throughout our work, we test the difference between distinct groups of patients, the observation independence assumption of the test is satisfied.

The second test used is the Kolmogorov-Smirnov test, the non-parametric test often used to determine if two samples have been drawn from the same distribution. This test is different from Mann-Whitney U test because it tests the difference in cumulative distributions (location and shape) of two samples whereas Mann-Whitney U test measures the discrepancy between the mean ranks of the groups. Currently, one drawback of using this method is that it can be affected by the presence of ties in the data (which are present in our dataset). Despite of this, the results obtained with this test closely follow those obtained with Mann-Whitney U test and the Anderson-Darling test.

Finally, the Anderson-Darling test can also be used to test if two samples are obtained from the same distribution. It has been shown [5] that this test is more powerful than Kolmogorov-Smirnov test to test normality. In addition, the corrections have been developed to rigorously test numerical data containing ties. Since many (especially biological) attributes have been transformed so that their value distribution closely resembles normal distribution, and given the presence of ties in our data, we believe adding this statistical test significantly increases the strength of evaluation of discovered findings.

b) To test the level and statistical significance of correlations between attributes we use Pearson [6] and Spearman's [7] correlation coefficient.

The Pearson's correlation coefficient is used to compute correlations between normally distributed pairs of attributes. If some attribute contains nominal values or at least one attribute is not normally distributed, we use Spearman's correlation coefficient. The main reasons for this is that Pearson's correlation can produce undesirable or misleading results if data is not normally distributed, as described in [8].

References

[1] Mann HB, Whitney DR. On a Test of Whether one of Two Random Variables is Stochastically Larger than the Other. Ann Math Statist. 1947;18(1):50–60. doi:10.1214/aoms/1177730491.

[2] Kolmogorov AN. Sulla Determinazione Empirica di una Legge di Distribuzione. Giornale dell'Istituto Italiano degli Attuari. 1933;4:83–91.

[3] Smirnov N. Table for Estimating the Goodness of Fit of Empirical Distributions. Ann Math Statist. 1948;19(2):279–281.

[4] Fritz W Scholz MAS. K-Sample Anderson–Darling Tests. Journal of the American Statistical Association. 1987;82(399):918–924.

[5] Stephens MA. EDF Statistics for Goodness of Fit and Some Comparisons. Journal of the American Statistical Association. 1974;69(347):730–737. doi:10.1080/01621459.1974.10480196.

[6] Pearson K. Note on regression and inheritance in the case of two parents. Proceedings of the Royal Society of London. 1895;58(347-352):240–242.

[7] Spearman C. The Proof and Measurement of Association Between Two Things. American Journal of Psychology. 1904;15:88–103.

[8] Hauke J, Kossowski T. Comparison of values of Pearson's and Spearman's correlation coefficients on the same sets of data. Quaestiones geographicae. 2011;30(2):87.
