# Supplementary material for: Using redescription mining to relate clinical and biological characteristics of cognitively impaired and Alzheimer’s disease patients
Source: PLoS One. 2017 Oct 31;12(10):e0187364. doi: 10.1371/journal.pone.0187364 (PMC5663625; doi:10.1371/journal.pone.0187364)
Supplement: S18 File — (PDF) [file pone.0187364.s018.pdf]

CLUS-RM algorithm pseudocode and the constraint-based redescription mining extensions when conjunction, negation and disjunction logical operators are used in redescription query construction

Matej Mihelčić, Tomislav Šmuc
Ruđer Bošković Institute, Zagreb, Croatia
{matej.mihelcic, tomislav.smuc}@irb.hr

Goran Šimić, Mirjana Babić Leko
Department for Neuroscience, Croatian Institute for Brain Research,
University of Zagreb Medical School, Zagreb, Croatia
{gsimic@hiim.hr,mbabic@hiim.hr}

Sašo Džeroski, Nada Lavrač
Jožef Stefan Institute, Ljubljana, Slovenia
{saso.dzeroski, nada.lavrac}@ijs.sl

In this document, we present the pseudocode of the CLUS-RM algorithm capable of using conjunction, disjunction and negation logical operators in redescription query construction. The query language used in the manuscript *Using Redescription Mining to Relate Clinical and Biological Characteristics of Cognitively Impaired and Alzheimer's Disease Patients* is constrained to only conjunction operators which also reflects on the presented, restricted pseudocode of the CLUS-RM algorithm. We also show how can constraint-based redescription mining extensions, introduced in the manuscript *Using Redescription Mining to Relate Clinical and Biological Characteristics of Cognitively Impaired and Alzheimer's Disease Patients* be used when conjunction, negation and disjunction logical operators are used in redescription query construction.

CLUS-RM algorithm description

The pseudocode of the CLUS-RM algorithm capable of using conjunction, disjunction and negation logical operator in redescription query construction is described in [1, 2] and presented in Algorithm 1.

Algorithm 1 The CLUS-RM algorithm

Require: First view (W_1), Second view (W_2), maxIter, Quality constraints $\mathcal{Q}$, OpSet
Ensure: A set of redescriptions $\mathcal{R}$

procedure CLUS-RM
  $[W_1^{(0)}, W_2^{(0)}] \leftarrow \text{createInitalData}(W_1,\ W_2)$
  $[P_{W_1^{(0)}}, P_{W_2^{(0)}}] \leftarrow \text{createInitialPCTs}(W_1^{(0)},\ W_2^{(0)})$
  $[r_{W_1^{(0)}}, r_{W_2^{(0)}}] \leftarrow \text{extractRulesFromPCT}(P_{W_1^{(0)}}, P_{W_2^{(0)}})$
  for Ind $\in \{1, \dots,\ \text{maxIter}\}$ **do**
    $[W_1^{(Ind)}, W_2^{(Ind)}] \leftarrow \text{constructTargets}(r_{W_1^{(Ind-1)}}, r_{W_2^{(Ind-1)}})$
    $[P_{W_1^{(Ind)}}, P_{W_2^{(Ind)}}] \leftarrow \text{createPCTs}(W_1^{(Ind)}, W_2^{(Ind)})$
    $[r_{W_1}^{(Ind)}, r_{W_2}^{(Ind)}] \leftarrow \text{extractRulesFromPCT}(P_{W_1^{(Ind)}}, P_{W_2^{(Ind)}})$
    if $\neg \notin$ OpSet **then**
      for $(R_{new} \in r_{W_1^{(Ind)}} \times_{\mathcal{Q}} r_{W_2^{(Ind-1)}} \cup r_{W_1^{(Ind-1)}} \times_{\mathcal{Q}} r_{W_2^{(Ind)}})$ **do**
        $\mathcal{R} \leftarrow \text{addReplaceDiscard}(R_{new},\ \mathcal{R})$
    else
      for $(R_{new} \in r_{W_1^{(Ind)}} \times_{\mathcal{Q}} r_{W_2^{(Ind-1)}} \cup r_{W_1^{(Ind-1)}} \times_{\mathcal{Q}} r_{W_2^{(Ind)}} \cup$
        $r_{W_1^{(Ind)}} \times_{\mathcal{Q}} \neg r_{W_2^{(Ind-1)}} \cup \neg r_{W_1^{(Ind-1)}} \times_{\mathcal{Q}} r_{W_2^{(Ind)}})$ **do**
        $\mathcal{R} \leftarrow \text{addReplaceDiscard}(R_{new},\ \mathcal{R})$
    if $\vee \in$ OpSet **then**
      for $R \in \mathcal{R}$ **do**
        if $\neg \notin$ OpSet **then**
          $r_1' \leftarrow argmax(R.maxRef(r_1),\ r_1 \in r_{W_1^{(ind)}})$
        else
          $r_1' \leftarrow argmax(R.maxRef(r_1),\ r_1 \in r_{W_1^{(ind)}} \cup \neg r_{W_1^{(ind)}})$
        $R_{ref} \leftarrow (r_1' \vee R.q_1 \times R.q_2)$
        if $\neg \notin$ OpSet **then**
          $r_2' \leftarrow argmax(R_{ref}.maxRef(r_2),\ r_2 \in r_{W_2^{(ind)}})$
        else
          $r_2' \leftarrow argmax(R_{ref}.maxRef(r_2),\ r_2 \in r_{W_2^{(ind)}} \cup \neg r_{W_2^{(ind)}})$
        $R_{ref} \leftarrow (R_{ref}.q_1 \times r_2' \vee R_{ref}.q_2)$
        $\mathcal{R} \leftarrow \text{addReplaceDiscard}(R_{ref},\ \mathcal{R})$
  $\mathcal{R} \leftarrow \text{minimizeQueries}(\mathcal{R})$
  return $\mathcal{R}$

The algorithm consists of four main parts: 1) Initialization, 2) Query creation (divided in query construction 2.1 and query exploration 2.2), 3) Redescription creation and 4) Redescription set optimisation.

1) In the initialization phase (line 2 in Algorithm 1), the algorithm makes a copy of each instance from the original dataset and shuffles the attribute values for the copies. For each attribute, the algorithm selects a random instance from the dataset and copies its value for the selected attribute to the target copy (value of one instance from the original dataset can be copied multiple times). This procedure breaks correlations between attributes in the copied instances. Each instance from the original dataset is assigned a target value 1.0 and each artificially created instance a target value 0.0. It is possible to use the PCT algorithm to create initial clusters, from such dataset, by distinguishing between original instances and copies containing shuffled values. The described procedure is repeated independently for both views contained in the dataset.

2.1) Each node in the obtained PCTs represents a cluster. These nodes are transformed to rules (line 4 in Algorithm 1) which are valid for the corresponding group of instances. More details about transforming PCTs to rules can be seen in [3].

2.2) The next step is to describe the same groups of instances, as those described by the produced rules, with the second attribute set (lines $6-8$ in Algorithm 1). To do this, for each instance of the original dataset, the algorithm constructs a set of target variables containing equal number of targets as number of rules constructed using the first set of attributes (for more details see [2]). The instance has a target value 1 on position j if it is described by the j-th rule from a set of rules constructed on the first set of attributes, otherwise the value is 0. Instances for which information is missing, making it impossible to determine the membership in support set of the query are also labelled with 0. We use the multi-target classification and regression capability of PCT to construct clusters on different views containing similar instances. The procedure is repeated by creating initial rules on the second view and describing similar sets of instances by using attributes from the first view.

3) Once the algorithm obtains rules for both views, it combines them by computing the Cartesian product of two rule-sets (depending on the allowed operators, line 10 or 13 in Algorithm 1). Notation $\neg r_{W_i}$ denotes a set $\{\neg r,\ r \in r_{W_i}\}$. Each redescription is evaluated with various user predefined constraints (such as minimal redescription accuracy, minimal support, maximal p-value, contained in a set of redescription quality constraints $\mathcal{Q}$), to select candidates for redescription set optimization. Lines 15-27 from Algorithm 1 allow CLUS-RM to produce redescriptions containing disjunction operator (if allowed by the user). Function $maxRef$ computes the improvement in redescription accuracy achieved after extending the redescription using disjunction operator. For more details on applying disjunction operator in CLUS-RM see [2].

Constraint-based redescription mining

The CLUS-RM algorithm incorporates constraints in redescription creation and one additional score in the optimization function used for redescription set creation.

To allow constraint-based redescription mining, we extend the CLUS-RM algorithm by adding a new set of constraints $\mathcal{C}$ containing the user-defined attributes of special interest and a type of CBRM used (parameter $\mathcal{T}$). Line 10 of Algorithm 1 is changed to $R_{new} \in (r_{W_1}^{(ind)})_{\{\mathcal{C},\mathcal{T}\}} \times_Q (r_{W_2}^{(ind)})_{\{\mathcal{C},\mathcal{T}\}}$. Analogous change is made in line 13 from Algorithm 1. Thus, redescriptions are created only by combining those queries that satisfy predefined constraints. For each redescription R_{new} we apply query minimization procedure before using redescription set optimization defined in lines 11 and 14 of Algorithm 1. If query minimization procedure removes any of the key constraint attributes defined in set $\mathcal{C}$ of CBRM, the created redescription is discarded.

In addition, CLUS-RM is extended with a new score measuring the overall score of a redescription satisfying user-defined attribute constraints. This score and its different variants are described in the main manuscript.

References

1. Mihelčić M, Džeroski S, Lavrač N, Šmuc T. Redescription mining with multi-label Predictive Clustering Trees. In: Proceedings of the 4th workshop on New Frontiers in Mining Complex Patterns. NFMCP '15. Porto, Portugal; 2015. p. 86–97. Available from: `http://www.di.uniba.it/~ceci/micFiles/NFMCP2015.pdf`.

2. Mihelčić M, Džeroski S, Lavrač N, Šmuc T. Redescription Mining with Multi-target Predictive Clustering Trees. In: Ceci M, Loglisci C, Manco G, Masciari E, Ras ZW, editors. New Frontiers in Mining Complex Patterns - 4th International Workshop, NFMCP 2015, Held in Conjunction with ECML-PKDD 2015, Porto, Portugal, Revised Selected Papers. vol. 9607 of Lecture Notes in Computer Science. Springer; 2015. p. 125–143. Available from: `http://dx.doi.org/10.1007/978-3-319-39315-5_9`.

3. Aho T, Ženko B, Džeroski S, Elomaa T. Multi-target Regression with Rule Ensembles. J Mach Learn Res. 2012;13(1):2367–2407.

4. Zaki MJ, Ramakrishnan N. Reasoning About Sets Using Redescription Mining. In: Proceedings of the 11th ACM SIGKDD International Conference on Knowledge Discovery in Data Mining. KDD '05. New York, NY, USA: ACM; 2005. p. 364–373. Available from: `http://doi.acm.org/10.1145/1081870.1081912`.
